# Cervical squamous cell carcinoma manifesting as a solitary metastasis to the skeletal muscle of the scapular region accompanied by invasion of the adjacent bone while on PD-1 inhibitor maintenance therapy: a case report

**DOI:** 10.3389/fonc.2026.1975605

**Published:** 2026-10-07

**Authors:** Na Zhang

**Affiliations:** 1 Department of Gynecologic Oncology, Chongqing University Cancer Hospital, Chongqing, China; 2 Chongqing Key Laboratory for the Mechanism and Intervention of Cancer Metastasis, Chongqing University Cancer Hospital, Chongqing, China

**Keywords:** cervical cancer, chemotherapy, programmed death-1 inhibitor, radiotherapy, skeletal muscle metastasis

## Abstract

Cervical cancer is a common malignancy in women worldwide, with distant metastases frequently occurring in the lungs, liver, bones, and lymph nodes. However, this case report presents an extremely rare isolated metastasis to the left scapular region,specifically located between the supraspinatus and the trapezius muscles, in a patient with locally advanced cervical squamous cell carcinoma who developed this lesion during maintenance therapy with a programmed death receptor-1 (PD-1) inhibitor after completing concurrent chemoradiotherapy. The clinical presentation of this metastatic lesion was atypical and posed a significant diagnostic challenge; however, multimodal imaging (MRI, PET/CT) and histopathological examination ultimately confirmed the diagnosis of cervical cancer metastasis. This finding underscores the necessity of maintaining a high level of vigilance for metastases in atypical sites, even in the era of immunotherapy. To manage this rare presentation, a multidisciplinary team developed a comprehensive treatment strategy centered on surgical resection, followed by postoperative adjuvant radiotherapy administered concurrently with chemotherapy and anti-angiogenic therapy(Q3W) consisting of paclitaxel 135 mg/m², cisplatin 50 mg/m², and bevacizumab 10 mg/kg. The patient remained recurrence-free at 13 months, suggesting that a multimodal combined local and systemic strategy may be feasible for selected patients with solitary muscle metastasis. This case not only adds to the clinical understanding of rare metastases in cervical cancer but also demonstrates that tumors can still metastasize to atypical sites under immunotherapy. These findings highlight the need for vigilance regarding metastases at atypical sites during immunotherapy. Any underlying mechanism—including immune editing and clonal evolution—remains speculative in the absence of paired molecular data and requires further validation.

## Introduction

Cervical cancer is the fourth most common malignancy among women globally, with squamous cell carcinoma as the predominant histological subtype (1). For locally advanced cervical cancer, exemplified by the Stage IIIB case presented herein, concurrent chemoradiotherapy is the standard treatment regimen. However, despite continuous advancements in therapeutic approaches, a considerable proportion of patients still remain at risk for recurrence and distant metastasis (1). Distant metastasis of cervical cancer typically exhibits a distinct pattern, with common sites including the lungs, liver, bone (especially the axial skeleton), and distant lymph nodes (2). Upon progression to the metastatic stage, treatment options become limited, and the efficacy of chemotherapy is often suboptimal, while immunotherapy and anti-angiogenic therapy present promising alternatives for select patients (3, 4).

Currently, the integration of PD-1 inhibitors into CCRT represents a pivotal advancement for high-risk locally advanced cervical cancer. The phase III KEYNOTE-A18 trial demonstrated that pembrolizumab plus CCRT followed by maintenance therapy significantly improves both progression-free survival (HR = 0.70, 95%CI 0.55–0.89) and overall survival (HR = 0.67, 95% CI 0.50–0.90) in this population. Building upon this paradigm, several PD-1 inhibitors—including sintilimab—have been extensively evaluated in LACC in China. Preliminary prospective data in 59 patients with node-positive LACC demonstrated that PD-1 inhibitor (camrelizumab or sintilimab) plus CCRT followed by maintenance achieved an ORR of 96.6% and a 1-year PFS rate of 85.3%, compared with 70.0% and 58.0% with CCRT alone. Consistent with this evidence, a registered clinical trial protocol (ChiCTR) specifically adopts the regimen of “sintilimab Q3W during CCRT plus sintilimab maintenance for 1 year” in FIGO 2018 IB3–IVA LACC. However, we acknowledge that sintilimab is not currently included in the officially approved indications for locally advanced cervical cancer, and that the globally recognized standard ICI for CCRT is pembrolizumab.

This case is unique as the patient developed metastasis at an extremely rare site—an isolated lesion in the skeletal muscle of the left scapular region (between the supraspinatus and trapezius muscles)—during Sintilimab maintenance therapy (a programmed cell death protein 1 [PD-1] inhibitor) following concurrent chemoradiotherapy. Although Sintilimab is not the globally standard regimen recommended by international guidelines, it was chosen based on regional drug availability and institutional protocols following multidisciplinary discussion and with the patient’s informed consent. Metastasis of cervical cancer to the skeletal muscle of the scapular region is rarely documented in the literature, rendering its clinical presentation easily mistaken for common musculoskeletal strains or primary soft tissue tumors, which often results in delayed diagnosis (5). The clinical value of this case lies primarily in its complete diagnostic evidence chain, established via multimodal imaging and precise pathological biopsy, which confirmed the metastaticsite,offering avaluable reference for the clinical identification of such rare metastases.

Secondly, this case underscores the need to reconsider the patterns of tumor progression in the era of immunotherapy. Metastatic progression was observed during maintenance treatment with a programmed death-1 inhibitor. While tumors can undergo clonal evolution via mechanisms such as immune editing, potentially resulting in oligometastasis or progression at non-canonical sites, such mechanisms remain speculative hypotheses in this case rather than conclusions drawn from our observations, and were not directly substantiated (6). Recent studies are increasingly reassessing the role of lymph nodes in tumor immunity, and the distant soft tissue and bone metastases reported in this case further broaden the current understanding of the complexity of tumor escape and metastatic site selection in the context of immunotherapy (6). Furthermore, the immune-checkpoint inhibitor-related thyroiditis presented in this case may also indicate a potential association between the patient’s distinct immune profile and metastatic progression, which warrants further investigation.

Finally, this case underscores the potential utility of a multidisciplinary treatment model in the management of complex and rare metastases. Following multidisciplinary team discussion, We adopted a comprehensive strategy centered on radical surgical resection, supplemented by postoperative adjuvant radiotherapy and systemic chemotherapy (paclitaxel, cisplatin, and bevacizumab), to address this solitary metastatic lesion. This approach proved feasible and was associated with favorable short-term disease control, although the contribution of each individual treatment component cannot be determined from a single case. This proactive approach of combining local and systemic therapy aligns with recent research directions exploring the integration of radiotherapy, immunotherapy, and chemotherapy to enhance the abscopal effects and improve the prognosis of advanced cervical cancer (7, 8). Therefore, this detailed case report not only expands the clinical spectrum of rare metastases in cervical cancer but also offers valuable insights into the individualized comprehensive management of patients with oligometastasis after immunotherapy.

## Case presentation

The patient was a 43-year-old female with a 12-year history of hyperthyroidism currently managed with stable pharmacological therapy (methimazole 5 mg daily). Baseline thyroid function tests in August 2023, before initiation of any anti-tumor therapy, showed a TSH of 2.13 μIU/mL (reference range 0.55–4.78 μIU/mL) and the free T4 of 1.10 ng/dL (reference range 0.8–1.8 ng/dL), both within normal limits. Thyroid autoantibodies were not assessed at baseline because the patient was clinically euthyroid and was asymptomatic for thyroid dysfunction.The patient’s family history and genetic history have no special additions.

In August 2023, the patient presented with dull lower abdominal pain. Initial cervical ThinPrep cytology test (TCT) showed no abnormalities, but *human papillomavirus* (HPV) testing was positive for type 16. In September 2023, the patient developed persistent right-sided low back pain. Computed tomography urography (CTU) indicated right hydronephrosis, thickening of the right ureteral wall at the intramural segment of the bladder, and ureteral dilation. The low back pain was initially attributed to lumbar muscle strain. In October 2023, pelvic MRI revealed an irregular cervical lesion with homogeneous signal intensity measuring approximately 1.0×1.3×1.3 cm, with apparent local involvement of the anterior vaginal fornix and findings suggestive of parametrial invasion. Gynecological examination conducted concurrently revealed thickening and shortening of the right parametrium extending to the pelvic sidewall. The pelvic lymph nodes exhibited mild enlargement and did not meet the radiological criteria for metastasis (short-axis diameter <10 mm); furthermore, no pathological confirmation was obtained. Pathological analysis of the colposcopy-guided biopsy confirmed invasive cervical carcinoma, which based on morphology and immunophenotyping, was consistent with poorly differentiated squamous cell carcinoma. The patient received a clinical diagnosis of cervical squamous cell carcinoma, staged as FIGO 2018 stage IIIB due to the presence of right hydronephrosis secondary to the tumor.

Given the high-risk profile of the disease (FIGO 2018 stage IIIB with tumor-related hydronephrosis and suspected parametrial invasion) and the substantial risk of distant metastasis after standard CCRT alone, the institutional MDT reviewed therapeutic options with the patient. After thorough consideration of emerging evidence supporting PD-1 inhibitor integration into CCRT for LACC—including the KEYNOTE-A18 trial with pembrolizumab and preliminary Chinese data on sintilimab—and given regional drug accessibility, the MDT proposed an exploratory regimen of sintilimab combined with CCRT followed by sintilimab maintenance. The patient provided written informed consent. We acknowledge that this regimen was off-label for sintilimab’s approved indications and was administered as an institutional MDT-sanctioned exploratory approach rather than as part of a formal clinical trial.

From October 13, 2023, to November 29, 2023, pelvic external beam radiotherapy was administered with a prescribed dose of 50.4 Gy in 28 fractions (1.8 Gy per fraction) to the planning target volume (PTV). Concurrent immunotherapy and chemotherapy were administered alongside radiotherapy, consisting of Sintilimab (200 mg every 3 weeks) and Cisplatin (40mg/m^2^ weekly). High-dose-rate brachytherapy was delivered in five fractions of 6 Gy each to the high-risk clinical target volume (HR-CTV D90) on November 2, 9, 16, 23, and 30, 2023. This regimen yielded a cumulative equivalent dose in 2Gy fractions (EQD2, α/β=10 Gy) of approximately 85–90 Gy to the HR-CTV. Individual dose-volume parameters for organs at risk were not routinely documented in the archived treatment records due to the retrospective nature of this report. Post-treatment pelvic MRI showed a significant reduction in the cervical lesion, indicating a partial response (PR) according to standard efficacy evaluation criteria. The patient received two cycles of sintilimab combined with paclitaxel and cisplatin on January 4 and January 25, 2024, followed by maintenance therapy with Sintilimab (200 mg, every 3 weeks) for 1 year. During the maintenance phase, the patient developed immune-related hypothyroidism and Hashimoto’s thyroiditis, which were managed with levothyroxine replacement therapy. The final administration of sintilimab occurred in January 2025. In early January 2025, while the patient was still undergoing sintilimab maintenance therapy, they developed left shoulder pain and discomfort of unknown etiology.

In January 2025, the patient developed left shoulder pain and discomfort of unknown etiology. On January 18, 2025, gynecological examination revealed right fornix adhesions and fusion of the cervix and fornix, resulting in the loss of the cervix’s normal anatomical contour;the uterus was mid-positioned and of normal size, while the parametrium showed thickening with poor elasticity on the rightside and fair elasticity on the left; no palpable abnormalities were found in the adnexa. Testing for HPV yielded negative results. Color Doppler ultrasonography revealeda hypoechoic mass measuring approximately 15.0×38.0×29.0mm located between the left supraspinatus and trapezius muscles. On January 21, 2025, shoulder MRI revealed a soft tissue mass between the left supraspinatus and trapezius muscles, measuring approximately 3.0×3.3×1.5cm, with T2 fat-suppression showing slightly hyperintense edges and central patchy hyperintensity, showing marked enhancement on post-contrast images with a central non-enhancing region, and indistinct borders with the left scapula, clavicle, supraspinatus, and trapezius muscles (**Figures 1A–C**). Thyroid function tests indicated the following: anti-thyroid peroxidase antibody >1300.00 IU/mL(reference range 0–60 IU/mL); anti-thyroglobulin antibody 10.00 IU/mL (reference range <4.5 IU/mL); thyroid-stimulating hormone 5.529μIU/mL (reference range 0.55-4.78μIU/mL).Regarding tumor markers, the Squamous Cell Carcinoma Antigen(SCCA) level was 1.30ng/ml(reference range 0-1.50 ng/ml), which is within normal limits. Laboratory tests revealed significantly elevated thyroid autoantibodies and increased TSH levels. In conjunction with the documented normal baseline thyroid function before treatment, these findings suggest immune-checkpoint inhibitor (ICI)-related hypothyroidism and Hashimoto’s thyroiditis, although the influence of the patient’s long-standing autoimmune thyroid disease cannot be entirely ruled out.

**Figure 1:**
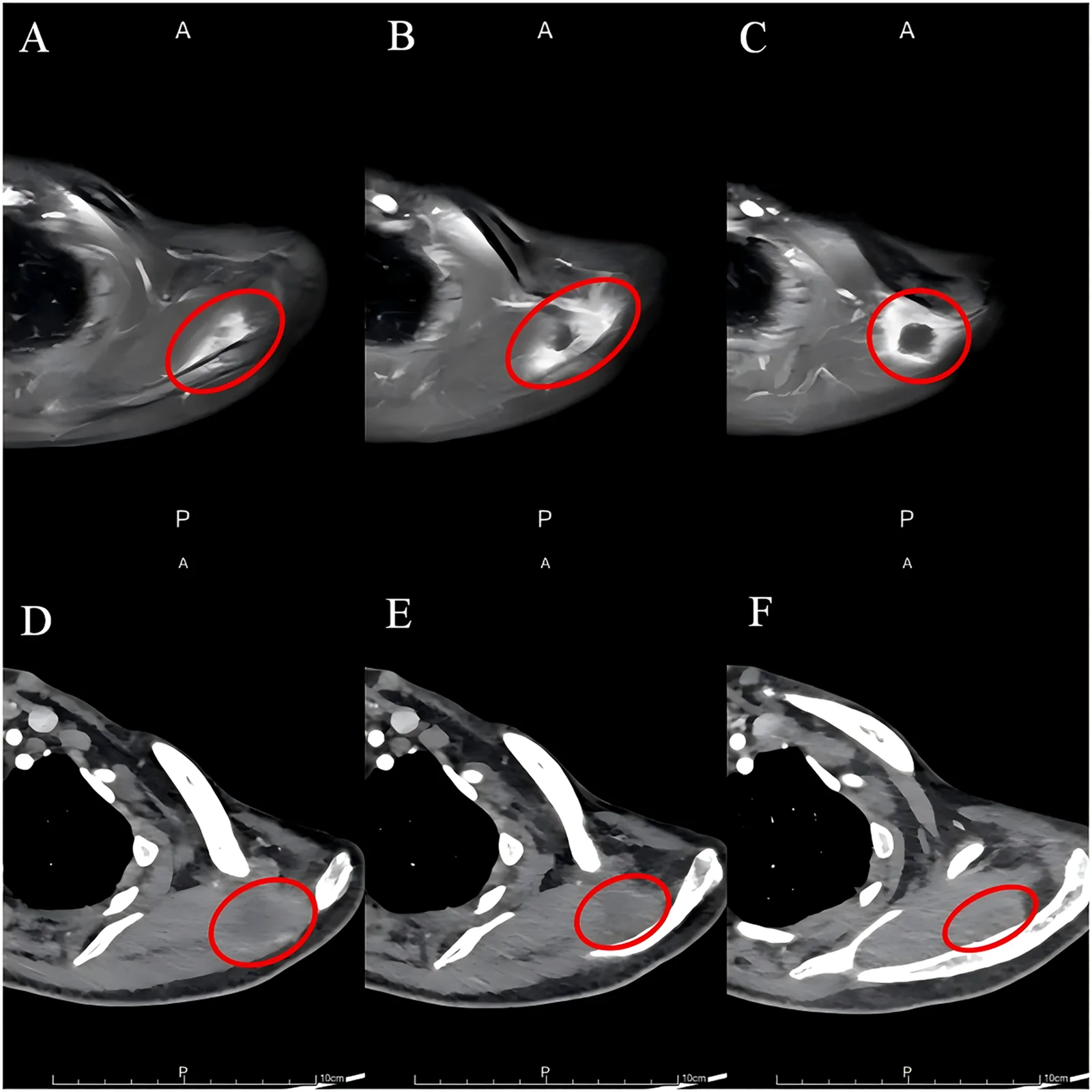
Pre-treatment MRI and CT images. Images **(A–C)** are axial MRI T2-weighted fat-suppressed sequences(imaging date: January 2025) demonstrating a soft tissue mass located between the left supraspinatus and trapezius muscles, measuring approximately 3.0 cm×3.3 cm×1.5 cm. The lesion shows ill-defined borders with the adjacent left scapula, clavicle, supraspinatus, and trapezius muscles, suggesting possible local invasion; metastatic malignancy could not be excluded. Images **(D–F)** are axial contrast-enhanced CT images(imaging date: February 2025) demonstrating a soft tissue mass located between the left supraspinatus and trapezius muscles adjacent to the left shoulder joint, measuring approximately 3.5 cm×2.5 cm×2.6 cm. The mass exhibits heterogeneous density with irregular, marked enhancement and ill-defined margins with the adjacent musculature, suggesting possible invasion; metastasis could not be excluded.The area indicated by the red circle shows the location of the lesion.

A needle biopsy of the shoulder mass was performed on February 6, 2025. Pathological examination of the lesion located between the left supraspinatus and trapezius muscles revealed atypical epithelial cell clusters within the fibrous stroma. When integrated with the patient’s history of cervical neoplasia, the morphometric analysis, and the immunohistochemical profile, the collective findings supported a diagnosis of p16-positive metastatic squamous cell carcinoma of cervical origin. Immunohistochemistry results: CK(Pan)(+), CK7(+), p16(+), p40(+), p53(approximately 10%+), CEA(+), ER(approximately 20%+), PR(-), HER-2(0), CDX2(-), Pax-8(-), Vimentin(-), Ki-67(approximately 40%+ in hot spots), TTF-1(-), S100(-), SOX-10(-).

On February 10, 2025, the HPV test was negative, squamous cell carcinoma antigen was 2.20ng/ml (Normal range:0-1.50ng/ml), and cytokeratin 19 fragment was 2.50ng/ml (Normal range:0-2.08ng/ml). On February 11, 2025, a whole-body PET/CT scan revealed a slightly hypodense mass between the left supraspinatus and trapezius muscles, measuring approximately 3.4×2.9 cm, with unevenly increased metabolism and an SUVmax of about 12.6, unclear boundary with adjacent muscles, and thinning of the adjacent left scapular bone cortex, consistent with a metastatic tumor (**Figures 2**, **3**). No additional abnormal FDG uptake suggestive of malignancy was observed in the cervix, pelvic lymph nodes, or any other distant site. Furthermore, the pelvic MRI performed on January 21, 2025 demonstrated no evidence of local cervical recurrence or suspicious pelvic lymphadenopathy. These findings confirmed that the left scapular-region lesion constituted a solitary oligometastatic recurrence. On February 13, 2025, CT imaging revealed a mass between the left supraspinatus and trapezius muscles adjacent to the left shoulder joint, measuring approximately 3.5×2.5×2.6cm, with heterogeneous density and irregular, marked enhancement on the contrast-enhanced scan, a central patchy non-enhancing area, ill-defined margins with the adjacent muscles, and no significant abnormal density or enhancement in the adjacent bone (**Figures 1D–F**). On February 17, 2025, Color Doppler ultrasound: A hypoechoic mass measuring approximately 17×28×39 mm was seen between the left supraspinatus and trapezius muscles, etiology undetermined; however, considering the clinical history, a metastatic lesion is suspected.

**Figure 2:**
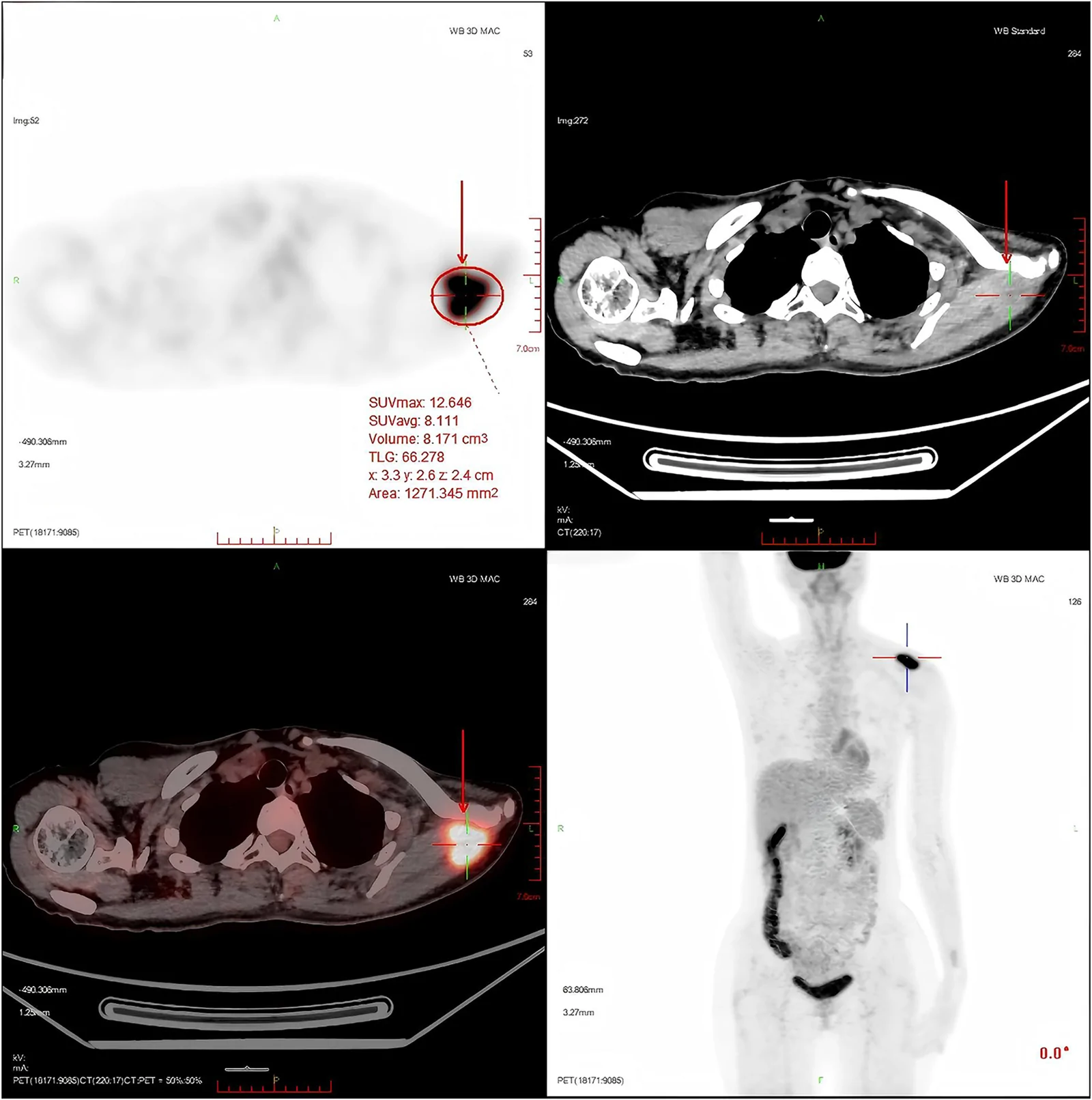
PET/CT imaging was performed in February 2025. A slightly hypodense mass is visible between the left supraspinatus and trapezius muscles, measuring approximately 3.4 cm×2.9 cm. The lesion exhibited heterogeneous internal density and ill-defined borders. It demonstrated heterogeneously increased metabolic activity with an SUVmax of approximately 12.6, showed indistinct demarcation from adjacent muscles, and was associated with thinning of the adjacent left scapular bone cortex. The area indicated by the red circle shows the location of the lesion.

**Figure 3:**
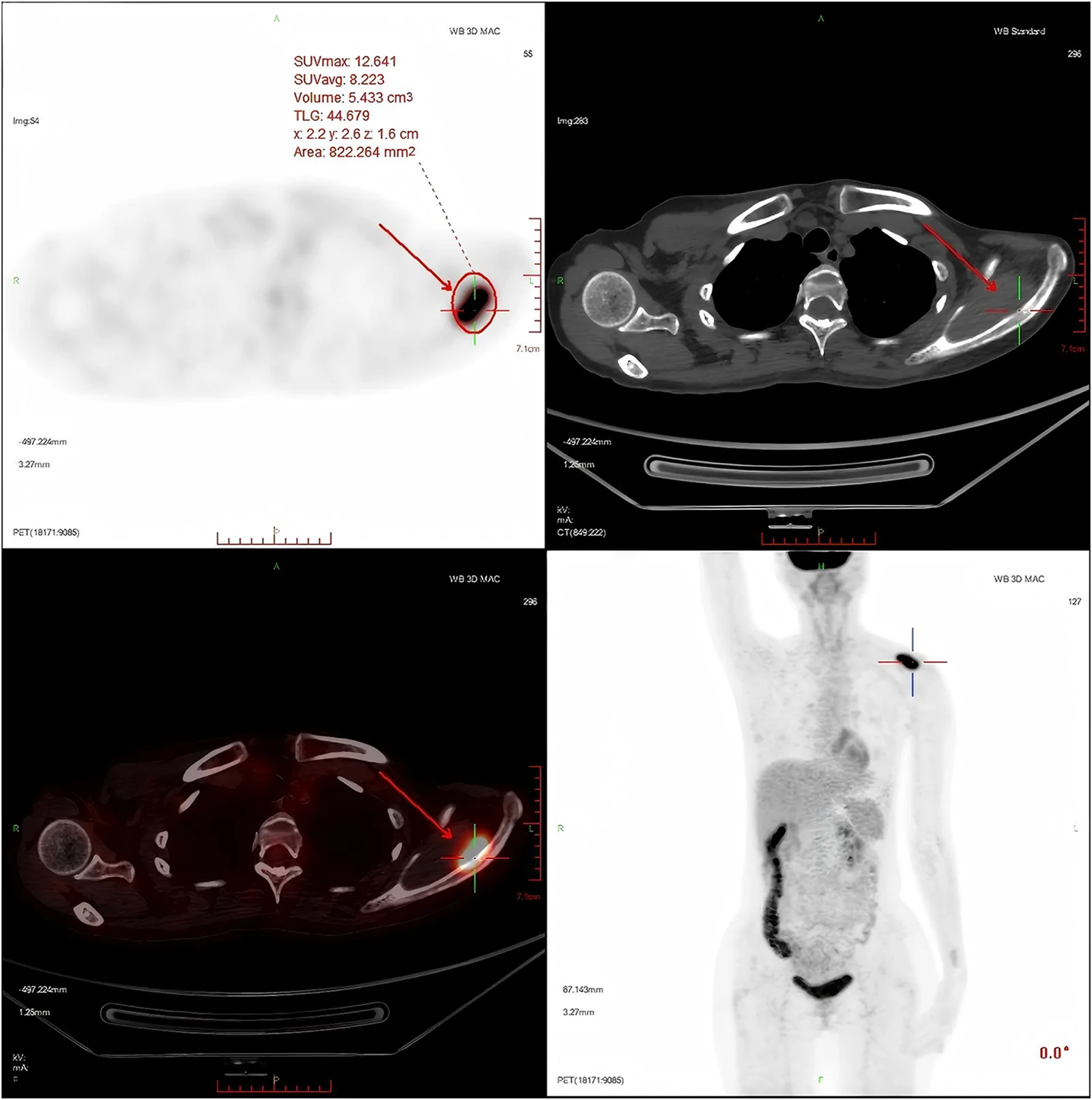
PET/CT imaging was performed in February 2025. A slightly hypodense mass is visible between the left supraspinatus and trapezius muscles, measuring approximately 3.4 cm×2.9 cm. The lesion exhibited heterogeneous internal density and ill-defined borders. It demonstrated heterogeneously increased metabolic activity with an SUVmax of approximately 12.6, showed indistinct demarcation from adjacent muscles, and was associated with thinning of the adjacent left scapular bone cortex. The area indicated by the red circle shows the location of the lesion.

After multidisciplinary team (MDT) discussion, the following diagnoses were established: 1. Cervical malignancy (squamous cell carcinoma, FIGO 2018 stage IIIB) post-chemoradiotherapy, with no evidence of local recurrence or residual pelvic disease at the time of the current presentation; 2. Solitary metastasis involving the skeletal muscle and adjacent bone of the left scapular region; 3. Immune checkpoint inhibitor (ICI-related) hypothyroidism. Whole-body PET/CT confirmed the absence of other distant metastases. MDT recommendations: 1. Perform comprehensive PD-L1 testing; 2. Following a comprehensive evaluation, proceed with either local surgery or local radiotherapy; 3. If surgery is not deemed feasible, administer a combination of local radiotherapy, bispecific antibody therapy,and TC chemotherapy.

On February 18, 2025, surgical resection was performed under general anesthesia for the resection of the metastatic tumor in the left scapular region. The procedure comprised a partial scapulectomy, including enbloc removal of the involved scapular spine, a portion of the adjacent scapular body, and the involved supraspinatus muscle, with a macroscopic safety margin of approximately 2 cm around the tumor. Intraoperatively, the tumor measured approximately 3.5cm×3.0cm×2.5cm. Preoperative imaging demonstrated ill-defined margins between the mass and the left scapula with cortical thinning, suggesting preoperative suspicion of bone involvement. Macroscopic complete resection, with no grossly visible residual disease. Postoperative pathology revealed atypical epithelial cell clusters within fibrous stroma. Based on clinical history, morphometry, and immunophenotype, these findings were consistent with p16-positive metastatic squamous cell carcinoma of cervical origin, showing invasion of the underlying muscle and bone. Immunohistochemical analysis of the mass in the left scapular region revealed the positive expression of CK(Pan), CK5/6, CK7, p40, and p16, along with a p53 positivity rate of 5% and a Ki-67 labeling index of 80%. These results support the diagnosis of metastatic squamous cell carcinoma of cervical origin with invasion of adjacent bone(**Figures 4A–C**). The Combined Positive Score(CPS) was 2 (**Figure 4D**).

**Figure 4:**
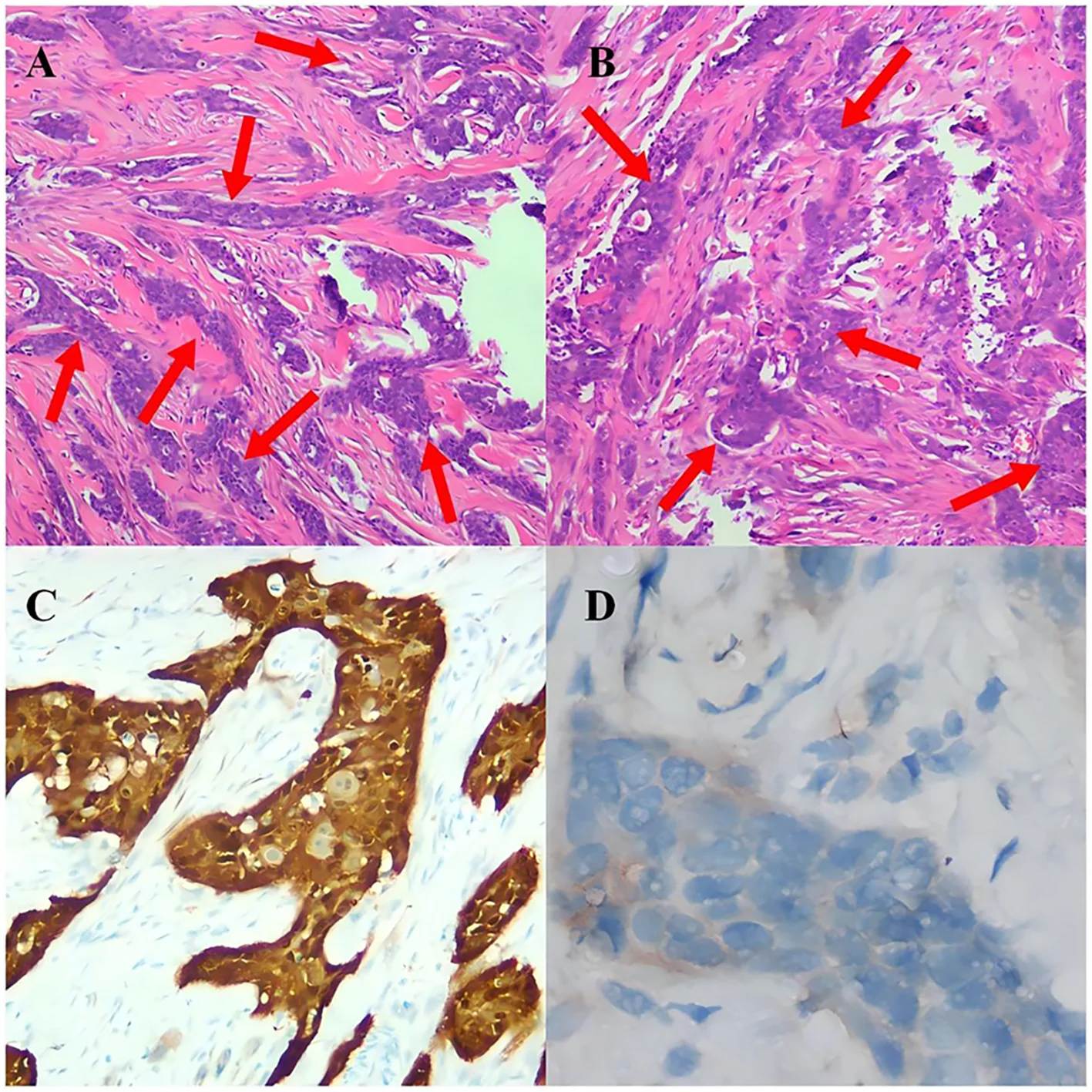
Postoperative pathological findings following partial scapulectomy of the left scapular region: **(A, B)** Display hematoxylin and eosin (H&E) staining at 100×magnification. Red arrows in the histological images indicate the lesion sites, while the purple areas represent squamous cell carcinoma. **(C)** shows the P16 immunohistochemical staining results (200×magnification); both the nuclei and cytoplasm exhibit brownish-yellow staining. **(D)** Displays the Programmed Death-Ligand 1 (PD-L1) expression was assessed by immunohistochemistry using the VENTANA PD-L1 (SP263) rabbit monoclonal antibody on a VENTANA BenchMark ULTRA platform. The combined positive score (CPS) was calculated as the number of PD-L1 stained cells (tumor cells, lymphocytes, and macrophages) at any intensity divided by the total number of viable tumor cells, multiplied by 100; the CPS of the metastatic lesion was 2.

Given the confirmed pathological invasion of the scapular bone, an resection with a macroscopically close margin (2 cm), and the high-risk nature of the disease, postoperative adjuvant radiotherapy was administered to reduce the risk of local recurrence. From March 20 to April 25, 2025, rotational intensity-modulated radiotherapy was administered to the metastatic tumor bed in the left shoulder, delivering a prescribed dose of 50 Gy in 25 fractions (2.0 Gy/F) to the planning target volume (PTV). During the same period, from March 18 to July 11, 2025, a total of 6 cycles of intravenous chemotherapy with Paclitaxel combined with Cisplatin and Bevacizumab (TP regimen+Bevacizumab) were completed: administered at doses of 190 mg (135 mg/m²) for Paclitaxel, 70 mg (50 mg/m²) for Cisplatin, and 500 mg (10 mg/kg, based on a body weight of approximately 50 kg) for Bevacizumab, given every 3 weeks (Q3W). The first 1–2 cycles of bevacizumab were administered concurrently with postoperative radiotherapy. This concurrent administration was deliberate, as combining bevacizumab with radiotherapy has been demonstrated to be feasible in cervical cancer without significantly increasing radiation-related toxicity. Bevacizumab was initiated only after the surgical wound had fully healed (maintaining a 4-week interval from surgery), thereby minimizing the risk of wound-healing complications. The patient was closely monitored for bleeding, wound-healing issues, and radiation recall reactions. No grade ≥3 bleeding events or wound-healing complications were observed. The main adverse event during chemoradiotherapy was grade III myelosuppression, which resolved following symptomatic treatment.

After completing all treatments, the patient was placed on a regular follow-up schedule. MRI and PET show localized absence of bone in the left scapular spine, with no new lesions in the surgical area of the left shoulder. No recurrent lesions are observed locally in the cervix, and no distant metastases are found elsewhere (**Figures 5**, **6**). As of this report’s preparation date, the patient has remained in stable condition for 13 months (**Figure 7**). Furthermore, tumor markers remained within normal or stable limits,and thyroid function was well-controlled with medication. The patient continues to be monitored in the outpatient setting.

**Figure 5:**
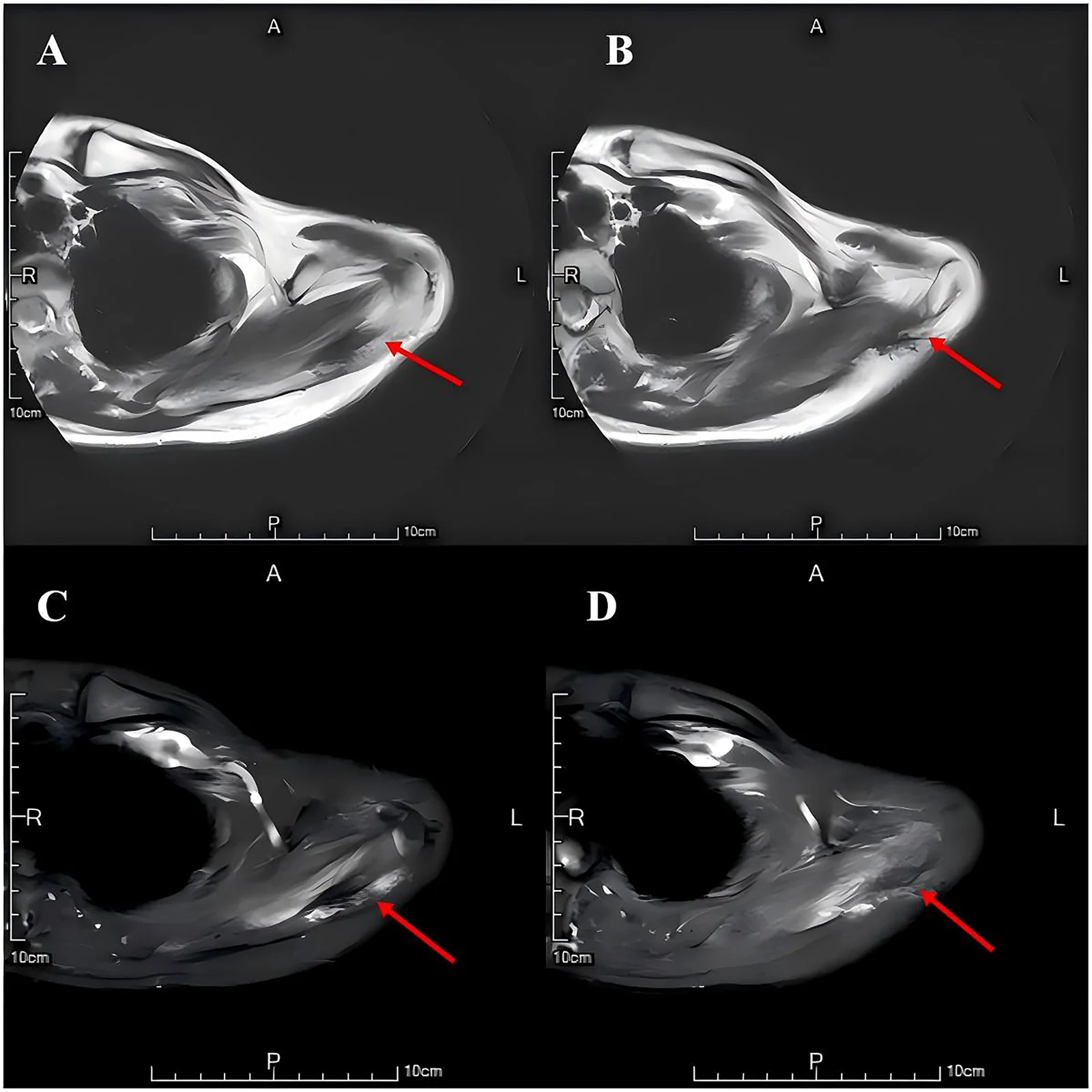
Axial MRI T2-weighted images (panels **A** and **B**) and fat-suppressed images (panels **C** and **D**) at 3 months post-treatment (imaging date: October 2025). After resection of the left shoulder mass, findings include a focalosseous defect of the left scapular spine (panels **A** and **C**), soft tissue swelling of the left shoulder with linear signal abnormalities,and adjacent subcutaneous exudative lesions (panels **B** and **D**), suggestive of postoperative changes.The area indicated by the red arrow represents the location of the left shoulder mass excision site.

**Figure 6:**
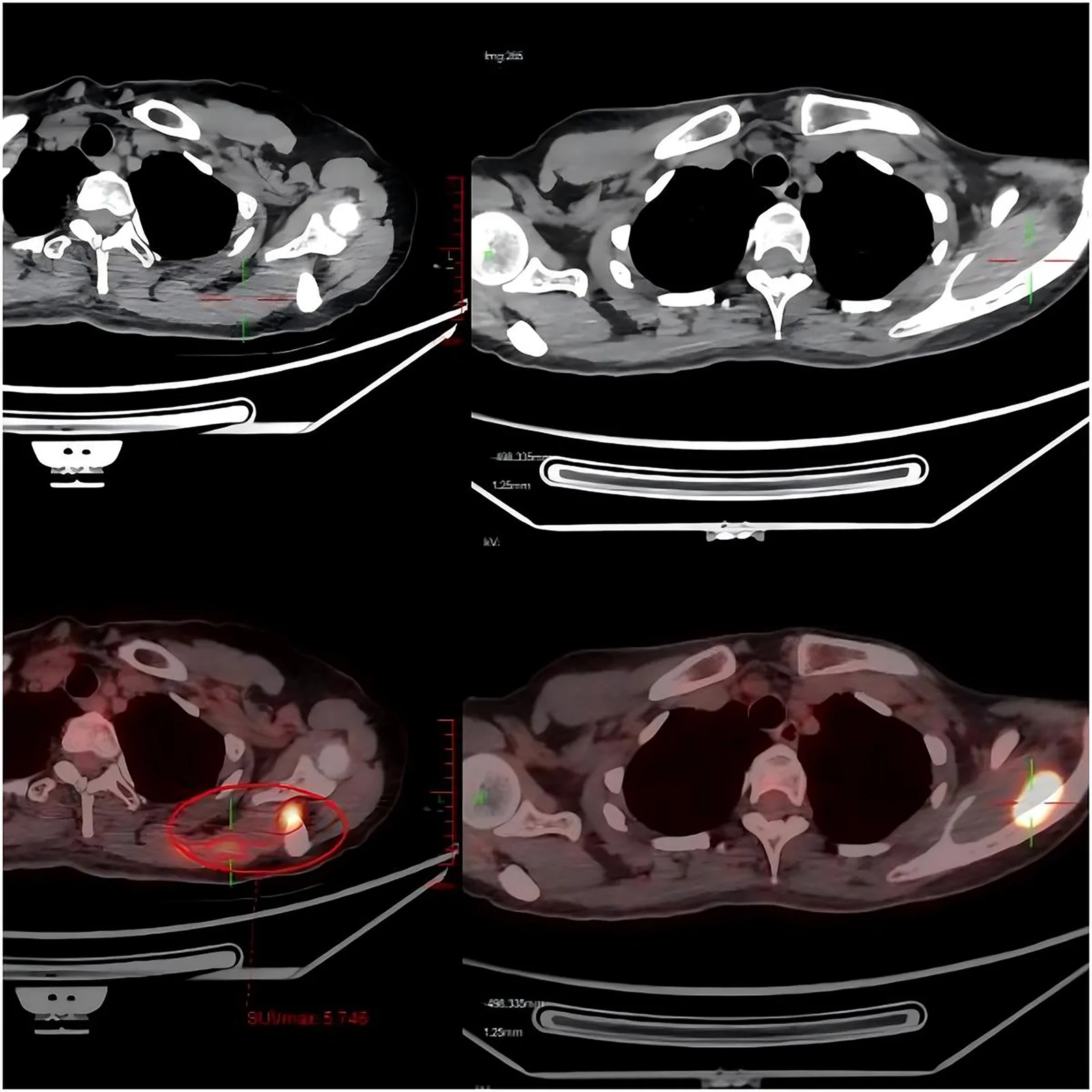
A PET/CT scan was performed 10 months post-treatment.In the postoperative left shoulder region, heterogeneous soft-tissue changes with mildly increased FDG uptake (SUVmax 5.7) were observed. This was interpreted as postoperative inflammatory change rather than residual tumor based on the following radiologic findings: the uptake was located in the operative bed with corresponding postoperative anatomic distortion; the degree of uptake had decreased substantially from the pre-treatment SUVmax of 12.6 on February 10, 2025, to 5.7; no discrete focal soft-tissue mass, nodularity, or marginal infiltration was identified to suggest a viable tumor deposit; and the accompanying CT findings were limited to subtle osseous density changes adjacent to the scapula, consistent with postoperative remodeling. The clinical and radiographic features were therefore judged most consistent with postsurgical inflammation. PET/CT did not reveal distant metastases.

**Figure 7:**
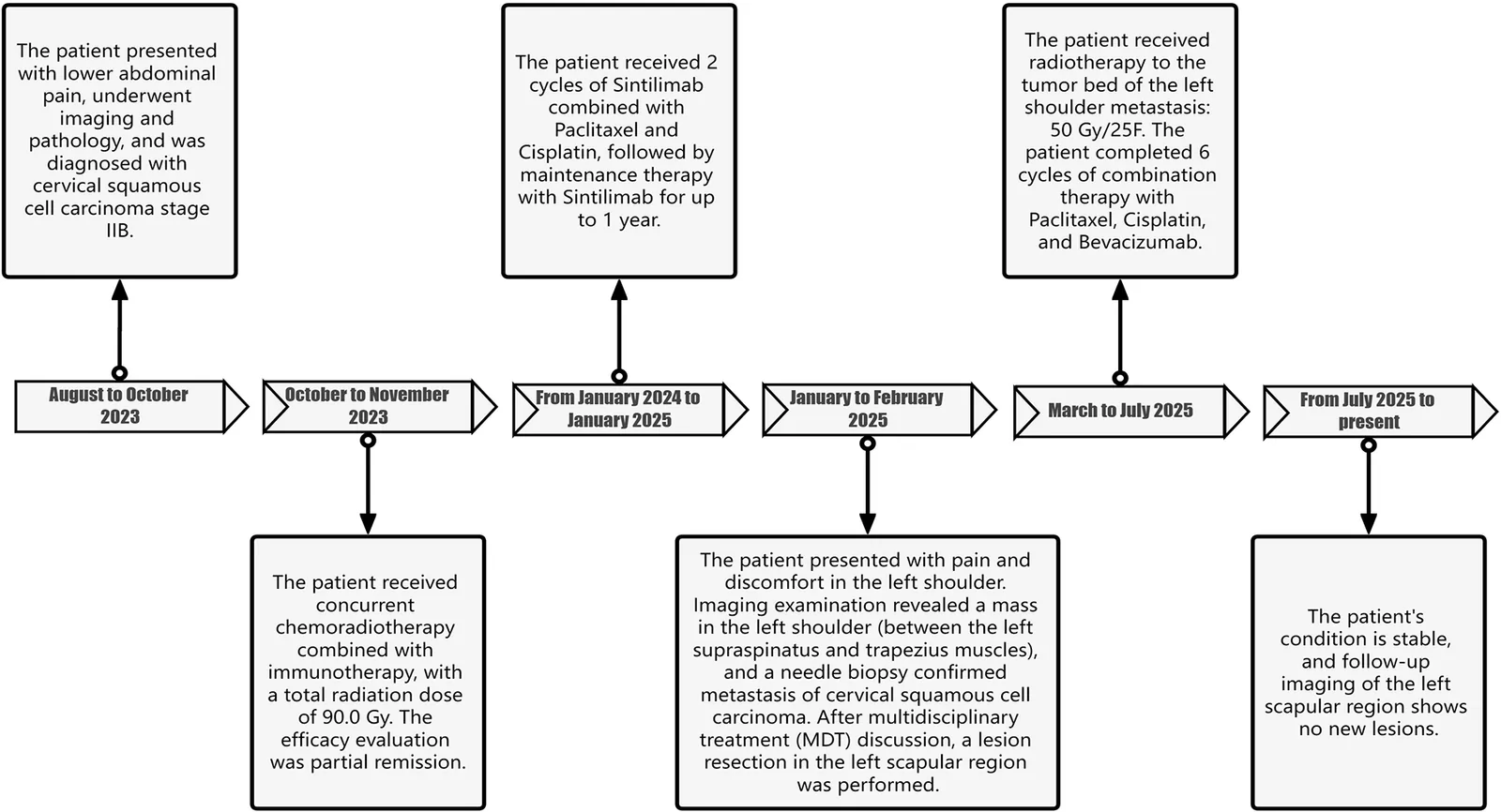
Timeline diagram of patient diagnosis and treatment. This image illustrates the process of the patient from initial treatment to recurrence and post-recurrence treatment, including time, diagnosis, important examination and test results, treatment plan, and evaluation of treatment outcomes.

## Discussion

Cervical cancer is the fourth most common malignancy among women worldwide, with approximately 604,000 new cases and 342,000 deaths in 2020 (9). Although the incidence has been reduced by screening and the widespread use of HPV vaccines, it remains a significant burden in developing countries (10). Cervical cancer spreads via direct extension, lymphatic dissemination, and hematogenous dissemination. The most common sites of distant metastasis are the lungs, liver, bones, and distant lymph nodes, whereas metastasis to skeletal muscle is exceptionally rare, with a reported incidence of less than 1% (11, 12).

This case report describes a 43-year-old patient with cervical squamous cell carcinoma (FIGO stage IIIB) who developed an isolated metastasis to the skeletal muscle of the left scapular region(between the supraspinatus and trapezius muscles) with secondary involvement of the scapula during maintenance therapy with a programmed cell death protein 1 (PD-1) inhibitor after radical concurrent chemoradiotherapy. This case is notable for the following reasons:

To contextualize the rarity of skeletal muscle metastasis in cervical cancer, we conducted a narrative literature review​ of previously reported cases. A comprehensive PubMed search was performed using the terms (“cervical cancer” OR “cervical carcinoma” OR “cervical squamous cell carcinoma”) AND (“skeletal muscle metastasis” OR “muscle metastasis” OR “soft tissue metastasis” OR “scapular metastasis”), covering the period from the database’s inception through January 2026. Given the rarity of the condition and the absence of a standardized classification, no formal eligibility criteria or systematic selection process were applied; cases were included based on clinical relevance and the availability of sufficient clinical details. This approach constitutes a narrative review rather than a systematic review, and the 31 cases summarized in **Table 1** are therefore illustrative rather than exhaustive.

**Table 1:** Summarizes our narrative review of 31 cases of skeletal muscle metastasis from cervical cancer, with key findings detailed below.

First Author	Publication Year	Age	FIGO Stage at Initial Diagnosis	Histological Type	Initial Treatment	PFS^a^	Site of Skeletal Muscle Metastasis	Metastasis at Other Sites	Symptoms of Skeletal Muscle Metastasis	Treatment After Recurrence	Survival
Mariya Y (41)	1990	NR^b^	NR	NR	NR	NR	Right biceps brachii	NR	NR	NR	NR
Schwartz LB (22)	1991	25	IIIB	Squamous cell carcinoma	Radiotherapy	Psoas major muscle metastasis at initial diagnosis	Right psoas major (abscess-like)	Bilateral external iliac and retroperitoneal lymph nodes	Right lower abdominal tenderness,inability to straight leg raise	Palliative Symptomatic Treatment	Death,5 months
Thomsen CF (30)	1991	65	IIA	Squamous cell carcinoma	Radiotherapy	6 years	Right deltoid	Suspicious vaginal fornix,bone metastases	Palpable mass only (no pain mentioned)	Surgery	Death,2 months
Singh GS (23)	1994	24	IIIB	Squamous cell carcinoma	Local palliative drainage+antibiotics	Psoas major muscle metastasis at initial diagnosis	Right psoas major	Retroperitoneal/pelvic cavity/abdomen lymph nodes+carcinomatous meningitis	None	Conservative Treatment	Death,4 to 5 months
George J (29)	1995	60	IIB	Squamous cell carcinoma	Radiotherapy	Approximately 8 years	Left psoas major region (lymph nodes encased)	L2/L3 osteolysis	Chronic back pain,mild paralysis of the left lower limb	Incision and drainage	Alive at report,OS NR
Wong BJF (37)	1995	34	IB	Small cellor neuroendocrine carcinoma	Surgery	24 months	Right masseter	None	Numbness in the right cheek+painless mass	Chemotherapy+Palliative Radiotherapy	NR
Kim CJ (18)	2001	63	II	Squamous cell carcinoma	Radiotherapy	Over 30 years	Right supraspinatus or infraspinatus (scapular region)	None	Painful mass in the right shoulder	Radiation therapy,wide excision+myocutaneous flap transplantation	Alive,12 months
Pathy S (19)	2002	38	IIIB	Squamous cell carcinoma	Radiotherapy	3 months	Right deltoid	None	Right shoulder pain+movement limitation	Palliative Radiotherapy	Alive at report(OS NR)
Devendra K (25)	2003	28	NR	Squamous cell carcinoma	Local palliative drainage+antibiotics	Metastasis to muscle at initial diagnosis	Left psoas major or iliacus region	Pelvic+inguinal lymph nodes	Pain in the left iliac fossa,left hip flexion	Drainage+Antibiotic+hospice	Lost to follow-up
Saadi I (28)	2003	62	IIB	Squamous cell carcinoma	Brachytherapy+surgery	5 years	Right psoas major	None	Pelvic pain+psoitis+right lower limb edema	Surgery+Radiotherapy	Alive,30 months
Kim HS (40)	2008	64	IB	Squamous cell carcinoma	Surgery+Adjuvant chemoradiotherapy	8 to 10 months	Right lower abdominal wall muscles	Recurrence in pericardium+anterior mediastinum+pelvic cavity	NR	Chemotherapy+radiation therapy	Alive,12 months
Padhi S (20)	2008	37	IIB	Squamous cell carcinoma	Concomitant chemoradiotherapy	21 months	Right deltoid (cyst-like)	None	Painless cystic swelling	Surgery+Local Radiotherapy	Alive at report(OS NR)
Kalra N (32)	2009	60	IIB	Squamous cell carcinoma	Radiotherapy	Approximately 24 months	Right psoas major+L2/L3 bone	L2/L3 osteolysis	Lumbago	Radiation therapy for metastases	Symptom improvement, OS NR
Karunanithi G (16)	2010	50	IIIB	Squamous cell carcinoma	Concomitant chemoradiotherapy	6 months	Left biceps brachii region	Multiple pulmonary metastasis	Painful swelling of the left arm	Local radiation therapy+chemotherapy	Alive at report
Ferreira CR (24)	2012	33	NR	Squamous cell carcinoma	No treatment	Systemic dissemination at first diagnosis	Left lateral femoral muscle	Thyroid,Mammary glands,Ovary,Adrenal glands,peritoneum,and multiple lymph nodes throughout the body	NR	None	Death,3 to 4 months
Sil AK (42)	2012	NR	NR	Adenocarcinoma	Surgery	4 months	Rectus abdominis sheath+ subcutaneous	NR	NR	Surgery+Chemoradiotherapy	Alive,5 months
Kalayci MU (14)	2013	48	NR	Squamous cell carcinoma	Surgery+Adjuvant chemoradiotherapy	34 months	Left rectus abdominis	None	Painful mass in the left abdominal wall	Wide excision+mesh repair+chemotherapy	Alive,3 years
Tamam MO (36)	2013	46	NR	Squamous cell carcinoma	Concomitant chemoradiotherapy	6 months	Left oblique abdominal muscle + right psoas major	Multiple subcutaneous nodules+residual cervix,iliac lymph nodes	NR	NR	NR
Basu S (39)	2014	52	IIIB	Squamous cell carcinoma	Concomitant chemoradiotherapy (without brachytherapy)	Approximately 2 to 3 years	Right psoas major	Prevertebral,left para-aortic,and bronchopulmonary hilar lymph nodes	Pain in both thighs	Chemotherapy+local radiation therapy	CR^c^, OS^d^ not reached
Chatzis P (35)	2014	40	IB1	Endometrioid adenocarcinoma	Surgery+Adjuvant radiotherapy	14 months	Right psoas major (abscess-like)	None	Low back pain,hip region pain,reduced movement in the right leg	Incision and drainage+excision+chemotherapy	Alive at report,OS NR
Kausar M (38)	2016	NR	IIIB	Squamous cell carcinoma	Concomitant chemoradiotherapy (without brachytherapy)	12 months	Left paraspinal muscles	Local recurrence of cervix+multiple pulmonary lesions	Swelling and pain in the left back	Refused treatment	Death due to disease (OS NR)
Skenderi F (15) (Case1)	2016	43	IIB	Squamous cell carcinoma	Concomitant chemoradiotherapy	24 months	Right scapular region or chest wall muscles	Initial solitary,posterior lung/pleura/axilla/skin	Painful mass in the right shoulder	Chemotherapy+Local Radiotherapy	Death,8 months
Skenderi F (15) (Case2)	2016	39	IB	Squamous cell carcinoma	Surgery+Adjuvant chemoradiotherapy	9 months	Right forearm muscles	Hepatic,retroperitoneal lymph nodes,multiple bone metastases	Painful mass in the right forearm	Surgery+Palliative Radiotherapy	Death,12 months
Albulescu DM (13)	2017	64	IB2	Adenosquamous carcinoma	Surgery+Adjuvant chemoradiotherapy	34 months	Left psoas major,iliacus,quadratus lumborum	None	Chest and low back pain	Chemotherapy (refused surgery)	Death occurred 11 months after muscle metastasis (total OS 45 months)
Varadarajan (34)	2017	51	IIIB	Squamous cell carcinoma	Chemoradiotherapy	24 months	Left deep to biceps brachii or brachialis	None	Pain and swelling in the left upper limb	Concomitant Chemoradiotherapy	Alive,24 months
Miyoshi A (21)	2019	57	IIB	Squamous cell carcinoma	Surgery+Adjuvant chemoradiotherapy	26 months	Right latissimus muscle,right biceps brachii	Initial solitary,posterior chest wall/pleura/mediastinum/ribs/T7 spinal canal	Initial asymptomatic,followed by pain+redness and swelling+ulceration	Surgery+radiation therapy+chemotherapy	Death,10 to 12 months
Rodriguez J (17)	2019	34	IIIB	Squamous cell carcinoma	Concomitant chemoradiotherapy (without brachytherapy)	21 months	Left T5–T8 paraspinal muscles	Right kidney	Painful mass between the left scapulae	Right Nephrectomy+Paravertebral Radiotherapy (Chemotherapy Refused)	Death,5 months
Omokawa, N (31)	2020	75	IB2	Adenocarcinoma	Surgery+Adjuvant chemotherapy	62 months	Right internal oblique muscle	None	painful mass in right lower abdomen	Surgery	Alive,10 months
Li D (33)	2021	51	NR	NR	Surgery+Adjuvant radiotherapy	24 months	Lower abdominal wall muscles	None	Hard mass with progressive enlargement (painless)	None	NR
Orellana T (27)	2022	56	IIB	Squamous cell carcinoma	Concomitant chemoradiotherapy	Initial PET scan showed muscle metastasis (misdiagnosed as inflammation)	Right proximal biceps brachii,axilla or chest wall	Right gluteal fat,coracoid bone	Right arm edema+pain	Chemotherapy+Palliative Radiotherapy	Home hospice(OS NR)
Santarelli IM (26)	2022	40	NR	Squamous cell carcinoma	Radiotherapy	Psoas major muscle metastasis at initial diagnosis	Left psoas major	None	Left groin pain,claudication,psoas sign	Local Radiotherapy	NR

PFS, Progression-Free Survival; NR, Not reported; CR, Complete response; OS, overall survival.

A narrative literature review of published cases of cervical cancer skeletal muscle metastasis up to 2026 (**Table 1**) revealed that none of the previously reported 31 cases were associated with immune checkpoint inhibitor (ICI) therapy. Initial treatments for these cases included surgery, radiotherapy, and chemotherapy; however, no patients had undergone immunotherapy (13–42). For example, Albulescu et al.(2017) reported a case of stage IB2 adenosquamous carcinoma with isolated left psoas major muscle metastasis 34 months after radical hysterectomy and adjuvant chemoradiotherapy (13). Similarly, Kalayci et al.(2013) reported a case of squamous cell carcinoma with metastasis to the left rectus abdominis muscle 34 months after radical surgery (14). Skenderi et al.(2016) reported two cases of squamous cell carcinoma with scapular and forearm muscle metastases 24 and 9 months after concomitant chemoradiotherapy, respectively (15). This case is notable for the emergence of isolated skeletal muscle metastasis during sintilimab maintenance—an off-label, MDT-sanctioned exploratory regimen modeled on the KEYNOTE-A18 paradigm. While the phase III KEYNOTE-A18 trial demonstrated significant PFS and OS benefits of pembrolizumab plus CCRT in high-risk LACC, the present case suggests that even under PD-1 blockade, tumors may still undergo clonal evolution and metastatic progression in atypical sites. However, it is important to recognize that the magnitude of benefit and resistance mechanisms may differ between pembrolizumab and sintilimab, and the experience with single-agent therapy reported here cannot be directly extrapolated to the globally established pembrolizumab-based standard of care.

This case is characterized by solitary oligometastasis in the appendicular skeletal muscle of the scapular region with involvement of the adjacent bone, rather than diffuse multi-organ metastasis. Among the 31 reported cases, 45.16% (14/31) were solitary skeletal muscle metastases, and 48.39% (15/31) were accompanied by metastases at other sites. For example, a case report by Karunanithi et al.(2010) described a patient with stage IIIB squamous cell carcinoma who developed metastasis to the left biceps muscle 6 months after pelvic chemoradiotherapy, concomitant with multiple lung metastases (16); a second case described by Rodriguez et al.(2019) involved a patient with the same histological subtype who developed metastasis to the left paravertebral muscle 21 months after concurrent chemoradiotherapy, as well as a metastasis to the right kidney. Among solitary metastases, cases involving both skeletal muscle and adjacent bone are particularly rare (17). Kim CJ et al.in 2001 reported a 63-year-old patient with stage IV cervical squamous cell carcinoma who developed metastasis to the right supraspinatus and infraspinatus muscles more than 30 years after intracavitary radiotherapy and external beam irradiation, a site similar to that in the present case; however, the lesion did not involve the scapula and occurred following conventional radiotherapy rather than immunotherapy (18). The oligometastatic feature of this case provides an opportunity for local treatment.

The patient initially presented with shoulder pain and a normal serum SCC-Ag level (1.30 ng/ml), and the diagnosis was ultimately confirmed via biopsy. This clinical course parallels several reported cases, including Pathy S et al. (19) ,who reported a patient with stage IIIB squamous cell carcinoma who developed right shoulder pain during radical radiotherapy, which was initially considered a benign muscle issue but was later confirmed by biopsy to be a metastasis to the right deltoid muscle; and Padhi S et al. (20), who reported a patient with stage IIB squamous cell carcinoma who developed painless cystic swelling in the right deltoid muscle 21 months after concurrent chemoradiotherapy, with delayed diagnosis due to atypical symptoms. These cases suggest that for patients with a history of cervical cancer, even with normal tumor markers, new and persistent musculoskeletal symptoms warrant a high index of suspicion for metastasis, necessitating timely multimodal imaging. In this case, the metastatic site demonstrated strong p16 positivity, a finding consistent with a cervical primary tumor, whereas PD-L1 expression was low (CPS = 2). None of the previous 31 cases documented PD-L1 expression status in metastatic sites, and this case is one of the few that document this parameter. However, given that PD-L1 testing was conducted exclusively on the metastatic lesion and no paired pre-treatment sample was available, this CPS value precludes interpretation as indicative of sensitivity or resistance to PD-1 blockade. Whether the low PD-L1 expression reflects an intrinsic property of the metastatic clone or a change induced by immunotherapy remains speculative and necessitates further elucidation.

We considered whether clonal evolution, immune editing, a low PD-L1 CPS of 2, or an immune-privileged skeletal-muscle niche contributed to progression during sintilimab. Because the primary tumour tissue was unavailable and no paired genomic, immunological, or longitudinal PD-L1 comparison was performed, these possibilities are presented solely as hypotheses to inform future research, not as demonstrated mechanisms of resistance in this patient (21).

What this case genuinely teaches the clinician is therefore practical: persistent, new focal musculoskeletal pain in a patient with cervical cancer warrants investigation even when tumour markers are normal and immunotherapy appears effective; rare skeletal-muscle metastases can mimic benign musculoskeletal disease; and tissue confirmation via image-guided biopsy with p16/CK7/p40 immunohistochemistry remains essential. Although the 13-month recurrence-free interval in this patient is promising and indicates the feasibility of the multimodal regimen and its association with short-term disease control, the single-case design precludes definitive conclusion regarding the contribution of individual treatment components or the overall efficacy of this strategy. The outcome should therefore be interpreted as hypothesis-generating rather than demonstrative of therapeutic superiority.

Regarding age at onset and staging, the patient ages ranged from 24 to 75 years, with a median of approximately 50 years. The patient in this case is 43 years old, which is slightly younger than the median. FIGO stages ranged from IB to IIIB, with stages IIB and IIIB being the most prevalent. This case is stage IIIB, consistent with most reports. Notably, in five cases, skeletal muscle metastasis was present at initial diagnosis, suggesting that some cervical cancers have the ability for hematogenous dissemination at an early stage (22, 23, 25–27).

Initial treatment: Most patients undergo radical hysterectomy, concurrent chemoradiotherapy, or multimodal therapy. In this context, concurrent chemoradiotherapy is the standard regimen. Notably, four cases developed skeletal muscle metastasis only after maintaining disease-free survival for over five years post-initial treatment, indicating that the risk of long-term recurrence cannot be ignored (18, 28–31).

Regarding time to metastasis, the interval from initial treatment to skeletal muscle metastasis exhibits considerable variability (from 2 months to over 30 years), with a median of approximately 25 months. In this case, the interval was 15 months, falling within the short-to-moderate range. This case developed metastatic disease during ICI maintenance therapy, whereas all other cases occurred after conventional treatment, underscoring its distinct clinical profile. The case reported by Kim CJ et al. in 2001 (18) presented with the most similar metastatic site (scapular muscle); however, the disease-free interval exceeded 30 years. Notably,the case reported by Omokawa N in 2020 exhibited a disease-free interval of 62 months, serving as aprime example of recurrence following an exceptionally long latency period (31).

Sites of metastasis: The most common site is the psoas major muscle (35.48%, 11/31), followed by upper limb muscles (25.81%, 8/31) and abdominal wall muscles (22.58%, 7/31). This case presents with metastasis in scapular region muscles (between the supraspinatus and trapezius muscles), which belong to the shoulder girdle muscle group. The high incidence of psoas major metastasis may be attributed to its rich blood supply and proximity to the pelvic cavity.

Metastasis pattern: 45.16% (14/31) were isolated, and 48.39% (15/31) were accompanied by metastases at other sites. This case exhibits dual involvement of “muscles and bone”, which is relatively rare. Kalra N et al. (32) reported a case of stage IIB squamous cell carcinoma with right psoas muscle metastasis and L2/L3 osteolytic bone metastasis 24 months after radical radiotherapy, showing a similar pattern. George J et al. (29) reported a case with left psoas muscle metastasis accompanied by L1 sclerosis, L2/L3 osteolysis, and left hydronephrosis 8 years after radiotherapy, also representing a combined “muscle and bone” metastasis pattern.

Clinical symptoms: Approximately 60% of patients present with pain as the primary symptom, and some may have a palpable mass. In the present case, shoulder pain was the initial symptom. Approximately 13.33% do not experience pain during the early stages and only present with a painless mass, indicating that pain alone is an insufficient criterion for diagnosis (20, 21, 33).

Treatment and prognosis: Treatment strategies encompass a wide range of modalities, including surgery, radiotherapy, chemotherapy, and combination therapy. Some patients with solitary metastasis may achieve long-term disease-free survival after undergoing aggressive local treatment. For example, Kalayci MU et al. (14) reported a case of solitary metastasis in the left rectus abdominis muscle that achieved 3 years of disease-free survival after wide resection combined with mesh repair and chemotherapy; Saadi I et al. (27) reported a case of solitary metastasis in the right psoas major muscle that achieved 30 months of local control after en bloc resection and postoperative radiotherapy; Varadarajan I et al. (34) reported a case of solitary metastasis in the left biceps brachii muscle that achieved 24 months of disease-free survival after local radiotherapy combined with concurrent cisplatin chemotherapy. Patients who did not receive local treatment or had extensive metastases generally had a poor prognosis: the case reported by Albulescu DM et al. (13), who refused surgery and received only chemotherapy, died 11 months after metastasis; the case reported by Rodriguez J et al. (17), who had renal metastasis and refused chemotherapy, died 5 months after recurrence; the two cases reported by Skenderi F et al. (15), both died 8–12 months after muscle metastasis. In this case, the patient received surgical resection, postoperative radiotherapy, chemotherapy, and targeted therapy, and remained recurrence-free after 13 months of follow-up. Although this follow-up period is encouraging and is comparable to several previously reported cases, it must be interpreted cautiously given the single-case design. These findings suggest that aggressive multimodal therapy may be a feasible option for selected patients with oligometastatic disease; however, its relative efficacy relative to other strategies cannot be established.

The diagnostic course of this case reveals several important pitfalls in diagnosing skeletal muscle metastasis from cervical cancer: (1) Misleading tumor markers: Normal SCC-Ag levels cannot rule out metastasis, especially in cases of isolated, low-volume metastatic disease; (2) Non-specific symptoms: Shoulder pain is common and requires differentiation from benign lesions; (3) Radiological misjudgment: Skeletal muscle metastasis may present with cystic changes or abscess-like alterations, which can be readily misdiagnosed as infectious processes (22, 35); (4) Cognitive blind spots in rare sites: The rarity of cervical cancer skeletal muscle metastasis leads to insufficient clinical awareness, causing these cases to be frequently overlooked during differential diagnosis. Devendra K et al (25). reported a case in which the left psoas muscle metastasis was initially misdiagnosed as an abscess and treated only with drainage; Santarelli IM et al (26). reported a case in which the left psoas muscle metastasis was treated with only local radiotherapy without systemic staging. For patients with a history of cervical cancer, new persistent musculoskeletal symptoms should warrant a high index of suspicion for metastasis, even with normal tumor markers. The combination of MRI and PET/CT is the preferred imaging strategy. Tamam MO et al (36). reported a case in which PET/CT simultaneously detected multiple muscle metastases in the left oblique abdominal muscles and right psoas muscle, along with subcutaneous nodules, highlighting the diagnostic utility of this modality. Suspicious lesions should undergo image-guided biopsy and subsequent testing for markers, including PD-L1 expression and HPV status. In this case, a definitive diagnosis was achieved via immunohistochemistry (showing strong p16 positivity, CK7+, and p40+), a finding consistent with the management approach for the majority of similar cases. Differential diagnoses include primary soft tissue sarcoma, lymphoma, metastatic carcinoma from distant primary sites, infections, and inflammatory diseases.

This case was managed with an individualized comprehensive treatment based on the principle of “oligometastasis”. Surgical resection established a complete pathological diagnosis, and postoperative adjuvant radiotherapy targeted minimal residual disease. The subsequent systemic therapy with paclitaxel, cisplatin, and bevacizumab was administered based on evidence from the GOG-240 study (43). Bevacizumab (10 mg/kg Q3W) was initiated after complete surgical wound healing and was strategically continued during the latter part of postoperative radiotherapy, a strategy supported by prior trials (e.g., RTOG 0417), which demonstrated the feasibility of concurrent bevacizumab and radiotherapy without unacceptable toxicity, contingent upon strict adherence to wound-healing precautions (44). After progression during ICI therapy, the treatment was switched to chemotherapy combined with anti-angiogenic therapy, accounting for immune resistance mechanisms. Historical data indicate that patients with solitary metastases who underwent surgical resection had better prognoses, while those who underwent no local intervention had poor outcomes, underscoring the efficacy of aggressive local therapy. Even during ICI maintenance therapy, tumors may still develop distant metastasis; clinicians should not place excessive reliance on the systemic protective effects of immunotherapy and should remain highly vigilant. The present case highlights the importance of maintaining heightened clinical suspicion for skeletal muscle metastasis in cervical cancer patients who develop new focal symptoms during immunotherapy, although intensified surveillance cannot be universally recommended based on a single case. Molecular testing of metastatic lesions, such as PD-L1 expression, tumor mutational burden(TMB), and microsatellite instability(MSI) status, may provide clinically relevant information in selected patients when it is likely to alter managemen; however, an unselected, systematic biopsy-based testing strategy is not supported by this case. The combined-modality approach integrating local treatment with systemic therapy was feasible and led to favorable short-term disease control in this patient. However, the relative contribution of each modality remains uncertain, and the superiority of this strategy over systemic therapy alone cannot be definitively concluded from this case report.The successful diagnosis and treatment of this case was facilitated by close collaboration among the departments of gynecologic oncology, orthopedics, radiation oncology, radiology, and pathology. For cervical cancer patients with rare metastatic patterns, MDT discussions facilitate the formulation of optimal comprehensive treatment plans, thereby mitigating the constraints inherent in a single-specialty approach.

This case report is subject to several limitations, as PD-L1 immunohistochemistry (CPS = 2) was performed solely on metastatic sites, precluding a comprehensive characterization of the immune microenvironment, including assessments of the density and composition of tumor-infiltrating lymphocytes (TILs), multiplex immunofluorescence labeling, and T-cell receptor (TCR) sequencing. Such data would be essential for elucidating the mechanisms underlying immunotherapy resistance. Given that the pathological specimens of the primary lesion are no longer available, we cannot perform paired genomic and immunomic comparative analyses between the primary and metastatic sites, and thus cannot directly elucidate the specific molecular mechanisms of clonal evolution and immune escape. At the time of the last follow-up (August 2026), the patient has been in a stable condition for 13 months. While these results are encouraging, long-term survival benefits warrant additional follow-up for confirmation. The data collection and analysis in this study employed a retrospective design, which may potentially introduce information bias and selection bias.

In the future, a multicenter international collaborative registry database should be established to systematically collect clinic opathological characteristics, treatment, and prognosis data of such cases. Future research can explore the following areas: (1) Using single-cell sequencing and spatial transcriptomics to analyze the immune microenvironment of metastases; (2) Exploring liquid biopsies such as ctDNA and exosomes for early prediction of resistance to immunotherapy; (3) Establishing PDX and organoid models for validation of drug resistance mechanisms and drug screening. For oligometastatic cervical cancer involving skeletal muscle, the optimal local treatment modality (surgery vs. SBRT), optimal systemic treatment regimen (chemotherapy vs. targeted therapy vs. immunotherapy), and treatment sequence remain unclear and necessitate investigation through prospective clinical trials in light of this case report, for patients progressing during ICI therapy, novel strategies such as combining other immune checkpoint inhibitors, immune agonists, epigenetic regulatory drugs, cell therapy, or tumor vaccines may be considered. Furthermore, advanced technologies such as whole-body diffusion-weighted imaging (WB-DWI) and artificial intelligence-assisted methods warrant investigation to increase the diagnostict yield for rate metastatic lesions.

## Conclusion

To our knowledge, this case is the first to report skeletal muscle metastasis of cervical cancer during ICI therapy. These findings demonstrate that distant metastasis may arise even during immunotherapy, underscoring the necessity of continued vigilance. The underlying mechanisms—including potential clonal evolution and immune escape—remain speculative and warrant further molecular and immunological elucidation. For patients with a history of cervical cancer, even if tumor markers are normal, new persistent musculoskeletal symptoms should raise a high suspicion of metastasis, necessitating timely multimodal imaging and histopathological confirmation via biopsy. Taken together, this case demonstrates that a multidisciplinary approach combining local and systemic therapies is a feasible treatment option for managing solitary skeletal muscle metastasis from cervical cancer. Upon disease progression during ICI therapy, transitioning to chemotherapy combined with anti-angiogenic therapy may be considered on a case-by-case basis; however, the contribution of each treatment component and the generalizability of this approach remain uncertain and should not be interpreted as proof of efficacy or an established standard of care. For patients presenting with skeletal muscle metastasis during immunotherapy, a multidisciplinary, individualized approach is recommended. In this case, a combination of surgical resection, postoperative radiotherapy, chemotherapy, and bevacizumab proved feasible and resulted in favorable short-term local and systemic control. Longer follow-up and further evidence are needed to validate its efficacy in this clinical context. Further evidence is needed to determine whether this strategy is optimal for other patients.
